# Particle exposure risk to a lavatory user after flushing a squat toilet

**DOI:** 10.1038/s41598-022-25106-4

**Published:** 2022-12-06

**Authors:** Tengfei (Tim) Zhang, Lifang Yao, Zilong Gao, Feng Wang

**Affiliations:** 1 School of Civil Engineering, Dalian University of Technology, Dalian, China; 2 Tianjin Laboratory of Indoor Air Environmental Quality Control, School of Environmental Science and Engineering, Tianjin University, Tianjin, China

**Keywords:** Environmental impact, Civil engineering, Health services

## Abstract

Squat toilets are widely used in developing countries due to local customs and low costs. The flushing of a squat toilet can entrain strong airflow and produce aerosols. This investigation constructed a lavatory mock-up with a squat toilet. The flushing-induced airflow was both visualized and quantitatively measured by particle image velocimetry. The maximum height of the impacted airflow was identified by an ultrasonic anemometer. For inference of the particle emission rate, the toilet bowl was covered by an enclosed box for particle concentration measurement. The risks from skin contact of the deposited particles on the flushing button and the door handle and the possible inhalation of the released aerosols were evaluated. The results revealed that flushing a squat toilet can drive toilet plume to rise up to 0.9 m above the toilet bowl. A single flushing process can produce 0.29 million particles with diameters greater than 0.3 μm, among which 90% of the particles are submicron-sized. The flushing may cause particles to deposit on the flushing button and lavatory door handle as well as inhalation exposure even remaining in the lavatory for half a minute after flushing, especially for those lavatory users whose respiratory zones are below 1.0 m.

## Introduction

Flushing a toilet can entrain airflow and produce droplets and droplet nuclei. The droplets and droplet nuclei may contain infectious microorganisms after an infector uses the toilet^1^. The detected SARS-CoV-2 in the urine and stool of infectors^2–5^ highlights the risk of transmission through the fecal–oral route. Early studies also reported the presence of SARS-CoV-1^6^, MERS-CoV^7^, norovirus and rotavirus^8^ in urine and feces. Even multiple flushes may not completely eliminate microorganisms from the toilet bowl surfaces^1,9,10^. The microorganisms may also form a biofilm on the inner wall of the toilet bowl^1^, and thus they may still be detected days or weeks later^11^. It is therefore crucial for the general public to understand the infectious transmission risk when accessing public lavatories and using toilets.

**Figure 1:**
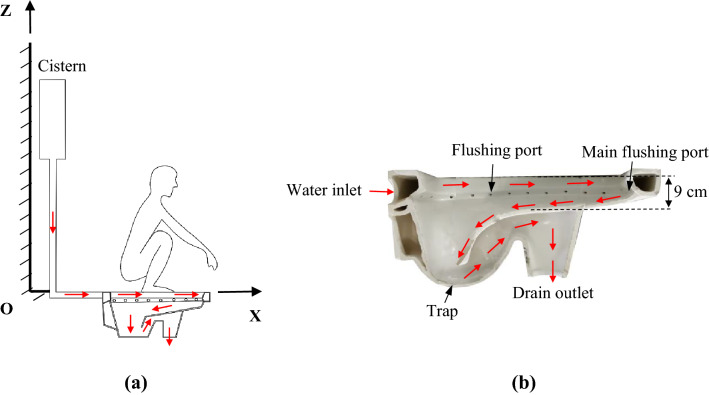
Schematics of a squat toilet system and water flow paths: (**a**) a user on the toilet, (**b**) sectional view of a realistic squat toilet and the path of flushing water flow.

For a typical squat toilet with a cistern as shown in Fig. 1, when the flush button is pressed, the water jets exit through many small ports and a main flushing port on one side of the toilet bowl. The flushing water collides with the inner surface of the toilet bowl, the excrement, and the mixture of excrement and flushing water. The flushing water rinses the toilet bowl, and then the flushing water together with the excrement is discharged into a sewer. Flushing a siphon toilet was claimed to generate strong airflow in the toilet bowl^12^. The pushing by high-speed air, the multiphase force action with the liquid and/or solid mixture, and shearing by the toilet bowl surfaces atomize the liquid and its mixture and produce droplets^13^. Droplets may be broken up when they are subjected to the shear force of the airflow^14^. Most of droplets quickly evaporate into the droplet nuclei. In addition, bubbles may be generated when air is entrained by stirred water, as in the case of air entrainment above seawater by wave action and whitecaps^15^. Bubble bursting may also produce aerosols^16^.

The atomization of aerosols is influenced by the toilet design and flushing energy^16,17^. For the same type of toilet, the greater the flushing energy, the more droplets and droplet nuclei are generated. A high-pressure toilet with a flushometer was reported to produce up to 0.28 million aerosols with diameters over 0.3 μm^17^. Despite similar flush volumes, the number of aerosols produced by the flushometer toilet was more than 3 times the number produced by a pressure-assisted gravity flow toilet^16^. The siphonic toilet produced approximately 1/14 the amount of bioaerosols as a wash-down pan for the same flush volume^18^. Compared to flushing without fecal waste, flushing with waste was claimed to increase the amount of bioaerosols, but did not much affect the total aerosols^19^. Large droplets were deposited within 1 to 2 m from the toilet^20^. Microorganisms were detected on frequently touched surfaces such as the toilet seat, lid, cistern, tap handle, lavatory door handle, and floor^21–24^. Note that the microorganisms on those surfaces may come from contaminated hands^25^ or from splashed or deposited droplets^21,26^. Therefore, there is a potential risk for subsequent lavatory users who touch the surfaces^27^.

The majority of the aerosols produced by toilet flushing were smaller than 3 μm in diameter^19^. Approximately 95% of the aerosols generated by flushing a siphon toilet were smaller than 2 μm, and 99% were smaller than 5 μm^16^. It was reported that the aerosols in the size range of 0.3–3 μm could be detected at a height of 1.52 m above the floor for at least 20 s after toilet flushing in a public lavatory^28^, and the number of the aerosols decreased with the height. When a vacuum toilet was flushed on a commercial aircraft, the concentration of aerosols in the breathing zone was measured with a peak rise of approximately 300 particles/L^13^. Bioaerosols could be detected at heights of up to 25 cm above a toilet seat within 90 min after flushing^29^. The presence of aerosols above the toilet was mainly ascribed to flows induced by the rising toilet plume. Toilet flushing could generate an updraft at least 1.0 m above the ground^12^. Flushing a vacuum toilet on a commercial aircraft even induced upward airflow from the toilet bowl to the breathing zone of a standing adult^13^.

In addition to the transient airflow induced by the flushing of a toilet, the lavatory ventilation could also affect aerosol transmission in the space. In a poorly ventilated lavatory, multiple flushes can lead to bioaerosol accumulation in the air^28^. In a compact lavatory with a ceiling-exhaust system, aerosols could be spread throughout the entire lavatory within 100 s after flushing^30^. A ceiling air supply together with a combined wall and floor exhaust ventilation system could remove bioaerosols effectively^31,32^. A ceiling air supply with an air exhaust on the rear wall even provided ISO-Class 5 cleanliness in a ward lavatory^33^. Suction vents on the bottoms of toilet seats were claimed to be effective in removing aerosols generated by toilet flushing^34^. Shortening the distance between the locations of the air exhaust and the toilet bowl was found to efficiently discharge bioaerosol particles^35^. To reduce bioaerosol dispersion, a viable approach was to flush the toilet with the lid closed when a toilet lid is present^18,28^.

The above review showed that toilet flushing can induce strong airflow and generate aerosols. Aerosols may carry infectious microorganisms and spread disease. Squat toilets are widely used in developing countries due to local customs and low costs. However, squat toilets use a large volume of water, and the toilet bowls are shallow. Flushing a squat toilet may produce a large quantity of aerosols. To the best of our knowledge, no previous study has systematically investigated squat toilets in terms of aerosol generation, transmission, and the resulting human exposure risk. The present investigation carried out measurements in order to fill this knowledge gap.

## Results

This section presents the measured airflow, particle concentration, and the possible exposure risk after flushing a squat toilet in a lavatory.

### Flow visualization

**Figure 2:**
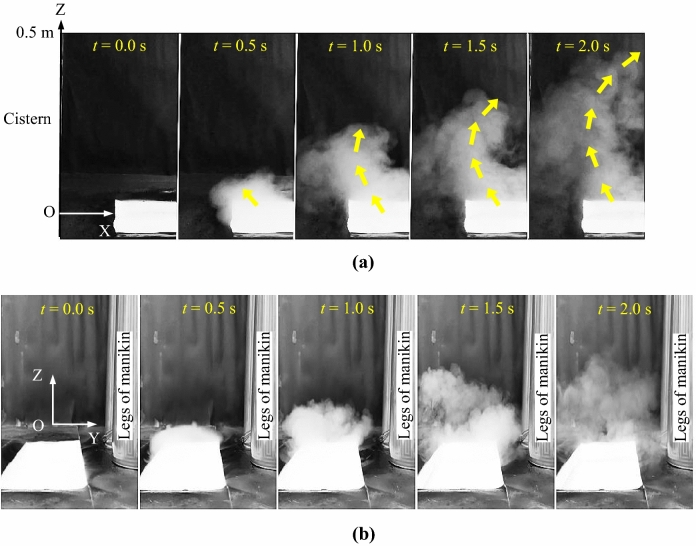
Visualization of transient airflow using water mist as a tracer in the lavatory during flushing: (**a**) lateral view, (**b**) front view.

Figure 2a shows the transient airflow of the flushing process at different times. The flushing button was pressed at *t* = 0 s, and the whole flushing process lasted four seconds. The water mist in the toilet bowl moved upwards towards the cistern at *t* = 0.5 s. The mist plume then continued to rise but moved back somewhat, i.e., to the right side of the figure, as shown at *t* = 1.0 s, 1.5 s, and 2.0 s. The visible water mist rose to approximately 0.5 m above the toilet. After *t* = 2.0 s, the water mist plume disappeared due to fast evaporation. Figure 2b shows the airflow viewed in the direction from the lavatory door to the cistern. The induced airflow was quite chaotic and of significant turbulence. Starting at *t* = 1.5 s, the water mist was in close proximity to the legs of the standing thermal manikin, indicating the possibility that infectious pathogens would be carried to the human body. More details of the visualized flows can be found from the supplemental videos (Supplementary files 1 to 2).

### Transient airflow field above toilet bowl

**Figure 3:**
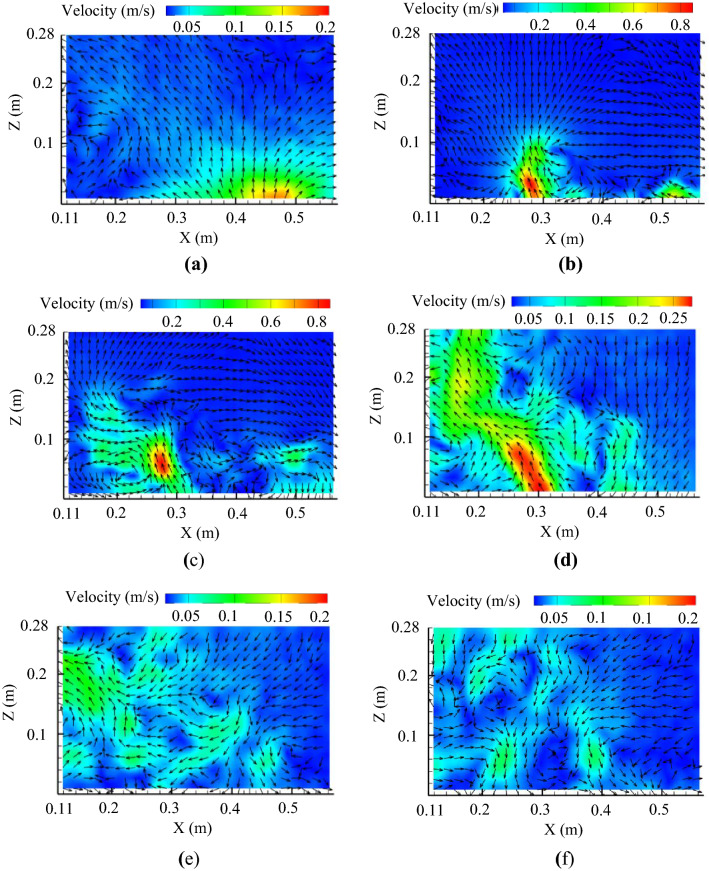
Measured transient air velocity distributions in the region of interest (ROI) after flushing toilet by PIV: (**a**) at *t* = 0.2 s, (**b**) at *t* = 0.5 s, (**c**) at *t* = 1.0 s, (**d**) at *t* = 2.0, (**e**) at *t* = 3.0 s, (**f**) at *t* = 4.0 s.

Figure 3 shows the distribution of air velocity in the region of interest (ROI) after flushing toilet by particle image velocimetry (PIV). Again, the flushing button was pressed at *t* = 0 s. At *t* = 0.2 s, the air was driven upwards generally by the water flow in the toilet bowl. The flow in the region with X < 0.45 m went left, while the flow in the region of X > 0.45 m went right, due to the shear by the water flow in the bowl. At *t* = 0.5 s, a strong updraft slightly to the left was formed, and the maximum velocity reached 0.8 m/s. The rising flow continued to develop at *t* = 1.0 s, and the updraft reached a height of 0.18 m. The maximum air velocity was approximately 0.6 m/s at *t* = 1.0 s. Meanwhile, some of the air in the bottom left corner was entrained, and a counterclockwise vortex was formed. At *t* = 2.0 s, the flow continued to rise, but the speed was reduced. Very clear downward flow in the region with X > 0.4 m can be observed. Starting at *t* = 3.0 s, as the flushing water flow decreased, the air movement above the toilet was very weak. The water flow had ceased completely at *t* = 4.0 s, at which time the weak air movement was still chaotic. During the entire flushing process, the measured maximum air velocity was 0.91 m/s at *t* = 0.488 s. More details of the transient airflow field can be found from the supplemental video (Supplementary file 3).

### Transient air velocities at typical locations

**Figure 4:**
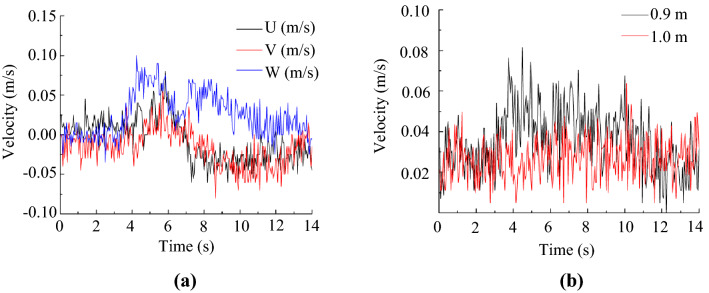
Measured transient air velocity profiles by an ultrasonic anemometer: (**a**) three velocity components at point P1 (0.3 m, 0, 0.7 m), (**b**) comparison of velocity magnitudes at points P2 (0.3 m, 0, 0.9 m) and P3 (0.3 m, 0, 1.0 m).

As the PIV system measured the airflow in a small region close to the toilet bowl, this investigation employed an ultrasonic anemometer to measure air velocities at some higher locations in the lavatory. Figure 4a illustrates the measured air velocities at point P1 (0.3 m, 0, 0.7 m). At 0.7 m above the floor, evident air velocities induced by the flushing could be sensed starting at *t* = 4 s. However, all the velocity components were quite low, with values less than 0.1 m/s. The vertical velocity component was slightly higher than the other two components. Figure 4b compares the omnidirectional velocity magnitudes at points P2 (0.3 m, 0, 0.9 m) and P3 (0.3 m, 0, 1.0 m). The velocity at a height of 0.9 m was slightly higher than that at a height of 1.0 m. The velocity at a height of 1.0 was almost unmeasurable, as it was quite close to the chaotic background value. The above results demonstrated that the flushing process may interrupt airflow at heights as high as 0.9–1.0 m.

### Particle source strength

**Figure 5:**
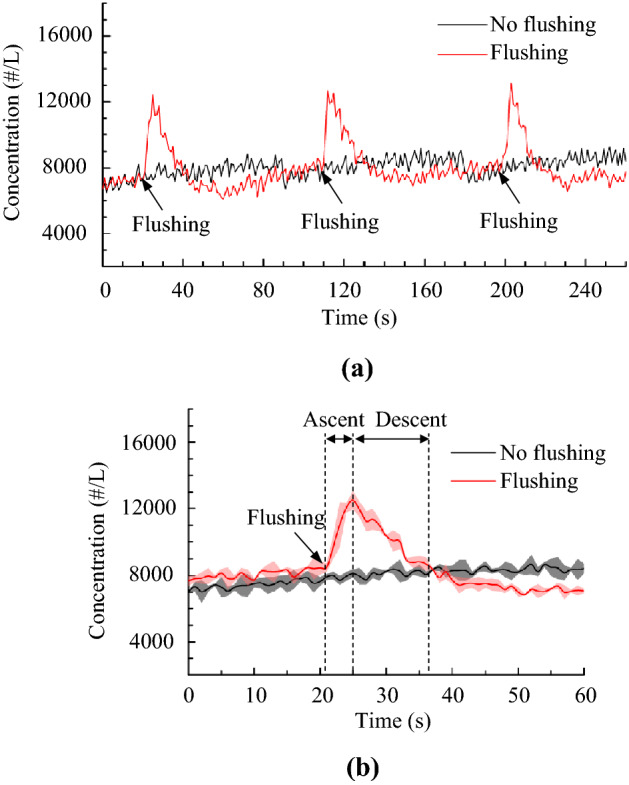
Measured temporal concentrations of particles with sizes ranging from 0.3 μm to 25 μm: (**a**) at the middle sampling port, (**b**) averaged particle concentrations at three sampling ports for three repeated flushing processes, where the shaded bands represent standard deviations.

Flushing toilet can generate numerous aerosols, as can be viewed from the supplemental video (Supplementary file 4). The particle concentrations inside the enclosed box above the toilet bowl were measured for inference of the total number of particles released during the flushing process. Figure 5a presents the measured total particle concentrations in a size range of 0.3–25 μm at the middle sampling port of the box. A total of three flushing operations were conducted, and the time interval between flushing operations was approximately 90 s. The background particle concentration without flushing was around 7,500 particles/L. After each flushing, the particle concentration increased to a peak of approximately 12,000 particles/L within 4 s. The concentration decayed to the background level in 10–13 s. The water flow and/or large water droplets helped deposit some particles after the flushing, which removed some airborne particles and resulted in a concentration below the background concentration of the no-flushing process. Figure 5b shows the averaged particle concentration at three sampling ports for three repeated flushing processes, where the error bands represent the standard deviation of the particle concentration. The small standard deviation implies that the mixing in the box and the repeatability of the flushing process were quite good.

Table 1 presents the generated particle numbers in each size bin and the proportion of the total particles inferred from the monitored particle concentrations. The number of generated particles decreased with the particle size. Approximately 74% of the particles were between 0.3 μm and 0.5 μm, and more than 90% of the particles were in the submicron range. The percentage of particles larger than 3 μm was less than 1%. The above particle size distribution was similar to that reported by Knowlton et al.^19^.

**Table 1:** Total and size-resolved particle numbers emitted from a flush of the squat toilet.

Particle size	Particle number and standard deviation	Proportion
0.3–0.5 μm	216,353 ± 20,399	73.8%
0.5–1.0 μm	51,099 ± 5528	17.4%
1.0–3.0 μm	21,860 ± 3412	7.5%
3.0–5.0 μm	1462 ± 219	0.5%
5.0–10.0 μm	52 ± 12	0.02%
10.0–25.0 μm	0	0
Total	293,237 ± 25,646	

### Fluorescent particles deposited on key surfaces

**Figure 6:**
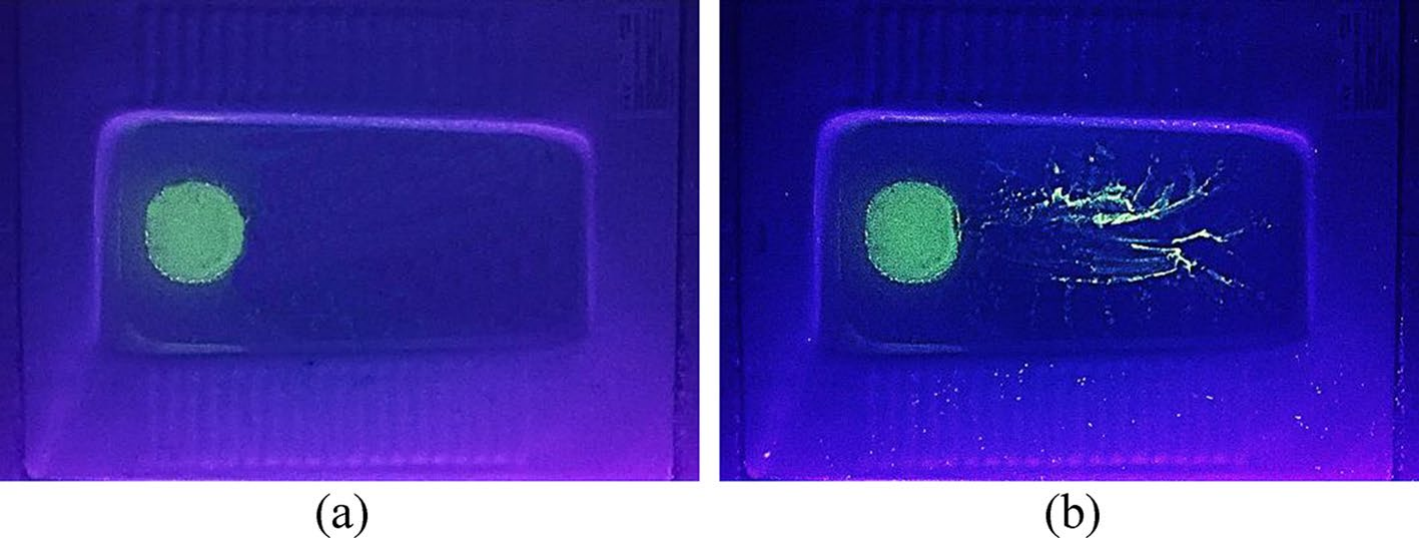
Deposition of fluorescent droplets/particles on the step area of the squat toilet: (**a**) before flushing, (**b**) after flushing.

The splashed droplets and deposition of airborne particles on some key surfaces were evaluated by means of the purposely released fluorescent powders to the cistern. Figure 6 shows deposition of the fluorescent particles on the step area of the squat toilet. There were no fluorescent particles deposited on this area before flushing, as shown in Fig. 6a. However, after flushing, a large number of fluorescent particles can be observed on the step area. Some of the fluorescent liquid can also be seen inside the toilet bowl. This implies that the splashed liquid and droplets carrying pathogens may settle on the step area of the squat toilet. A lavatory user’s shoes may come into contact with the pathogens and carry them to other indoor floors. Some of the pathogens may become airborne if the pathogens deposited on floors are resuspended by a disturbance.

**Figure 7:**
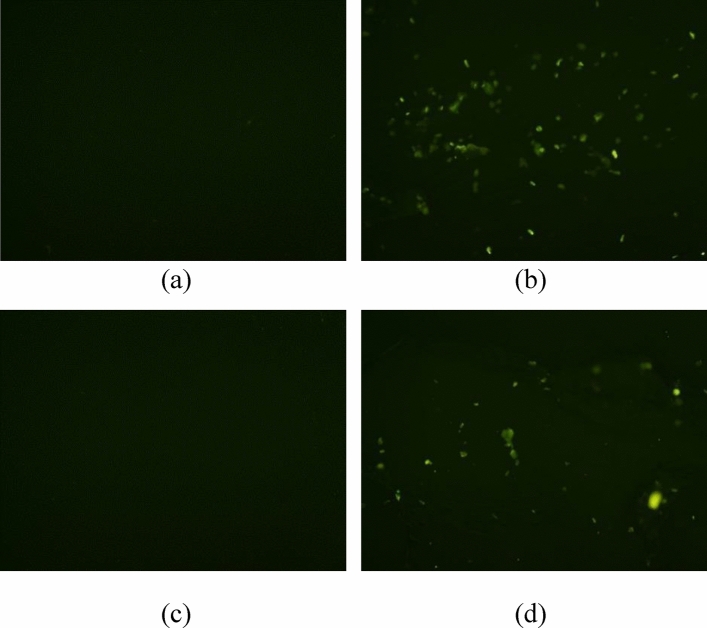
Deposition of fluorescent particles on the flushing button and lavatory door handle after flushing of the toilet: (**a**) on the flushing button without addition of fluorescent powder to the cistern, (**b**) on the flushing button after addition of fluorescent powder to the cistern, (**c**) on the door handle without addition of fluorescent powder to the cistern, (d) on the door handle after addition of fluorescent powder to the cistern.

Figure 7 shows the deposition of fluorescent particles on the flushing button and the lavatory door handle. When the toilet was flushed without the addition of fluorescent powder to the cistern, no fluorescent particles were observable, as shown in Fig. 7a,c. In contrast, flushing with the fluorescent powder in the cistern caused significant deposition of fluorescent particles on both the button and door handle, as shown in Fig. 7b,d, respectively. This implies that a lavatory user’s hands may become contaminated by contact with both the flushing button and the lavatory door handle.

### Exposure evaluated by means of a tracer-gas concentration

**Figure 8:**
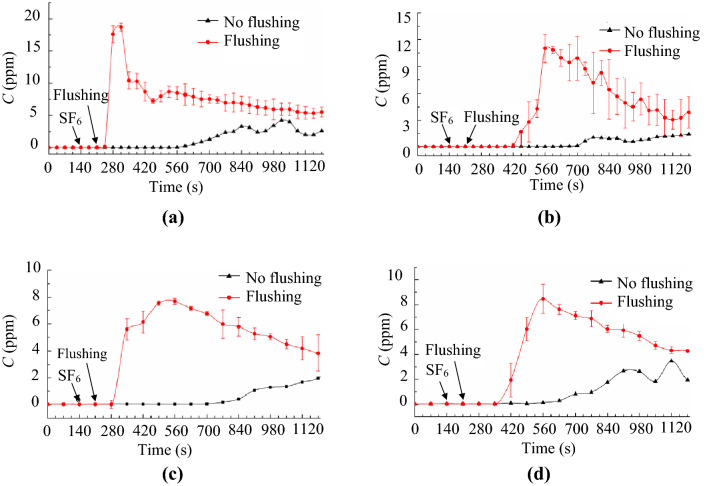
Transient SF_6_ concentrations in breathing zone after flushing of the toilet: (**a**) at point #1 (0.4 m, 0.15 m, 1 m), (**b**) at point #2 (0.4 m, 0.15 m, 1.5 m), (**c**) at point #3 (0.7 m, 0, 1 m), (**d**) at point #4 (0.7 m, 0, 1.5 m).

Due to interference by the huge number of airborne particles in the background air, it would have been extremely difficult to measure the particles purely released from the toilet bowl. Instead, this investigation measured the concentration of SF_6_ tracer gas to evaluate the possible human inhalation exposure. Figure 8 shows the transient SF_6_ concentrations at four points. The error bars in the figure indicate the standard deviation for three repeated tests. The background concentration of SF_6_ was approximately 0.02 ppm. The release of SF_6_ began at *t* = 140 s, and the flushing was implemented at *t* = 210 s. Due to diffusion, the SF_6_ gas spread at a slow rate if the toilet was not flushed and if there was no disturbance in the lavatory. It would take 13 to 15 min for the concentration to rise significantly. Once the toilet has been flushed, the SF_6_ gas in the squat toilet bowl was carried upward by the airflow and reached point #1 within 30 s, as shown in Fig. 8a. In contrast, it would take 3–4 min for the concentration to rise at point #2 after flushing of the toilet. This shows that at a low breathing height, such as that for children, the inhalation risk would be greater. Similar results were obtained at points #3 and #4 in front of the toilet. However, the flushing-induced peak concentrations at points #3 and #4 were lower than those at points #1 and #2. This finding indicates that the farther away a user is from the trap, the lower the inhalation exposure risk.

### Projected particle exposure to inhalation

**Figure 9:**
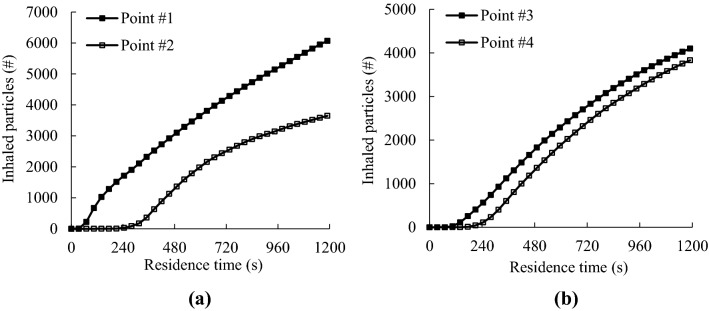
Averaged inhaled particle numbers for different residence times after flushing of the toilet when no face mask was worn: (**a**) at point #1 (0.4 m, 0.15 m, 1 m) and point #2 (0.4 m, 0.15 m, 1.5 m), (**b**) at point #3 (0.7 m, 0, 1 m) and point #4 (0.7 m, 0, 1.5 m).

The total inhaled SF_6_ tracer gas of a lavatory user can be obtained by integrating the monitored SF_6_ gas concentration with the residence time after flushing of the toilet and multiplying with the pulmonary ventilation rate. Because the totally released SF_6_ gas into the toilet bowl was known, the inhaled ratio defined as the percentage of the inhaled SF_6_ gas with respect to the totally released SF_6_ gas could be calculated. Suppose the generated droplet nuclei would well trace the SF_6_ gas, the inhaled ratio of the SF_6_ gas would be identical to that of the droplet nuclei. Figure 9 presents the inhaled particle numbers varying with the residence time after flushing of the toilet. The inhaled particle number was simply the product of the averaged, totally generated particle number shown in Table 1 and the inhaled ratio of the SF_6_ gas. If the breathing zone of a lavatory user was at point #1, the user might inhale more than 6,000 particles generated from the toilet bowl for a residence time of 20 min when no face mask was worn. The residence for even half a minute could inhale several particles and more than 200 particles for a minute. The maximum inhaled particle number was less than 4,200 at points #2 to #4. The inhaled particles were close to zero for the residence of the initial 2 min at point #3, and for the initial 4 min at points #2 and #4. It clearly indicated that the breathing zone far away from the trap and at a higher height could effectively reduce the inhaled particle number.

## Discussion

For the sake of simplicity, this investigation did not consider a realistic flush with feces in the toilet bowl. Notably, in a sitting toilet, the presence of fecal waste has not been found to increase the generated aerosol number, although the bioaerosol number might be greater when feces are present^19^. The inferred number of particles emitted per flushing in this investigation was slightly greater than the reported 287,400 in a sitting toilet with a high-pressure flushometer of 400 kPa^17^. However, according to another study, the flushing of a sitting toilet with a flushometer > 350 kPa reportedly generated 145,000 particles^16^. The discrepancy among may be due to the different operating conditions of the tested toilets and the measurement methods. Nevertheless, it is certain that the squat toilet produced a larger number of aerosols per flush than the sitting toilet.

A laser-based particle counter was used to measure the particle concentrations in this investigation. The counting efficiency of the particle counter was 100% for particles larger than 0.45 μm, but only 50% for 0.3 μm particles. Thus, the number of aerosols with particle sizes smaller than 0.3 μm could not be measured. In the future, a condensation particle counter could be employed to measure the number of fine particles smaller than 0.3 μm. Considering that the aerodynamic diameter of most viruses is less than 0.1 μm, as is the case with SARS-CoV-2, it would be of value to measure particles smaller than 0.3 μm. In addition, the flushing would increase the relative humidity inside the enclosed box above the toilet bowl. The flushing generated droplets first and then most droplets evaporated into droplet nuclei. It is believed that both the number and sizes of droplet nuclei may vary with the relative humidity. Variation of the measured particle concentrations and their spectrums with the relative humidity awaits further investigation.

This paper did not directly measure the aerosols in breathing zone; instead, a tracer gas was measured. The reason was that the background air had a high particle concentration, and as such it was difficult to differentiate generated particles from background particles. Alternatively, an analysis to the inhaled particle exposure was conducted by projecting the generated particles inside the toilet bowl into the breathing zone, based on the same inhaled ratio between the SF_6_ tracer gas and the generated particles. Note that the SF_6_ gas is heavier than the air and the aerosols are in a discrete phase. Future researchers may consider measuring the droplet nuclei directly in breathing zones. In a previous study, a generated particle number of 8,498 was reported to cause a concentration peak in the breathing zone in the lavatory of a commercial aircraft^13^, inside which the background particle concentration was at least two orders of magnitude lower than that in this investigation. A future investigation could measure the particle concentration by putting the lavatory mockup into a cleanroom and assess the associated particle exposure directly. The projected inhaled particle number in this investigation did not consider the intake efficiency of airborne particles into the respiratory tracts and the capture of some particles by nose hair or other mechanisms.

The PIV system was used to measure the airflow without disruption. However, there were still uncertainties in the measured results. Our further analysis (see Supplementary file 5) shows that the uncertainties of the measured air velocities were mostly in the range of 0.02–0.05 m/s. The largest uncertainty was less than 0.08 m/s. Note that PIV can only provide the airflow field in a relatively small region of interest. The PIV system used in this investigation was only two-dimensional, so that the velocity component in the third dimension was not measured. The flushing-induced three-dimensional airflow could also be studied further.

## Conclusions

This investigation measured the airflow and particle concentrations, inferred the number of generated particles, and analyzed both the surface contact and the inhalation exposure risk after the flushing of a squat toilet in lavatory. Based on the obtained results, the following conclusions are drawn:

- Flushing a squat toilet can induce strong water flow in the toilet bowl and entrain transient airflow above the toilet bowl. The measured maximum air velocity is 0.91 m/s near the trap 0.5 s after flushing of the toilet. The airflow becomes very weak 4 s after the toilet flushing. The flushing-induced plume flow may reach a height of up to 0.9 m.
- Flushing a squat toilet can generate a significant number of droplets and their nuclei. A single flushing process produces 0.29 million particles that are larger than 0.3 μm, among which 90% of the particles are submicron and 74% of the particles range from 0.3 to 0.5 μm.
- For lavatory users whose respiratory zones are below 1.0 m, especially for children, there is a high risk of inhalation exposure, even when the user remains in the lavatory for half a minute after flushing. The farther away a user is from the trap, the lower the inhalation exposure risk. In addition, the flushing may cause particles from the toilet bowl to deposit on both the lavatory door handle and the flushing button, and the resulting surface contact risk merits attention.

## Materials and methods

### Lavatory mock-up

Squat toilets involve a wide variety of designs, and the gravity-flow (“cistern”) system and pressure-valve (“flushometer”) system are the two most common types. In a cistern system, water flows under gravity, and thus water pressure is nearly irrelevant to the flushing process. Therefore, a cistern system was chosen in this study, as shown in Fig. 1. During flushing, the water jets exit through 35 ports, each with a diameter of 8 mm. There is also a main rectangular flushing port of 5 cm × 1.5 cm located on the right side of the toilet bowl. The bowl of the squat toilet is shallow with a 9 cm depth in the middle.

**Figure 10:**
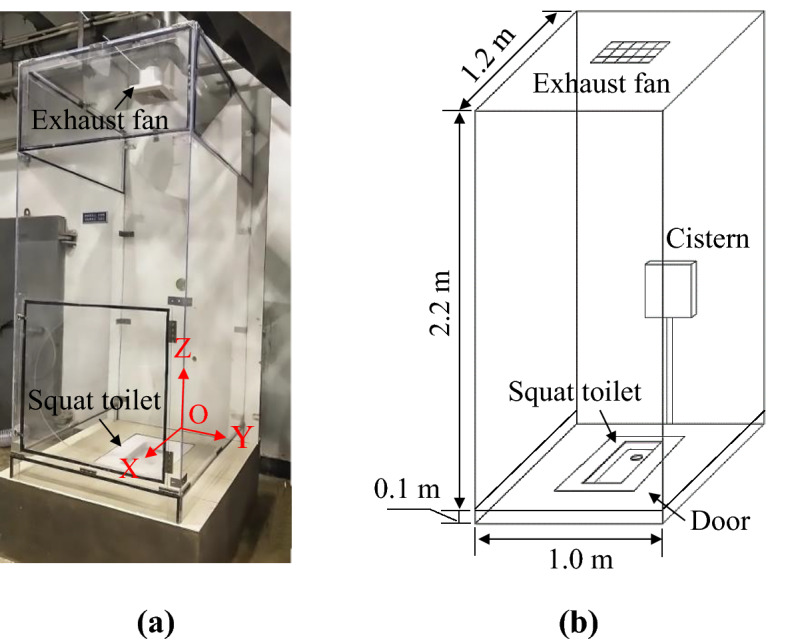
A lavatory mockup with a squat toilet inside: (**a**) photograph of the lavatory, (**b**) schematic of the lavatory.

The squat toilet was located in a lavatory mockup with dimensions of 1.2 m × 1.0 m × 2.3 m, as shown in Fig. 10. For visualization of the airflow inside, the front and side lavatory walls were constructed from transparent acrylic panels, while the rear wall was a solid wood panel. The floor was paved with tiles. An exhaust fan with a constant flow rate of 120 m^3^/h was installed on the lavatory ceiling. The cistern had a flushing capacity of 6.0 L, and the top of the cistern was 1.0 m above the floor. To avoid disturbance of the air inside the lavatory, the cistern was located outside the lavatory.

**Figure 11:**
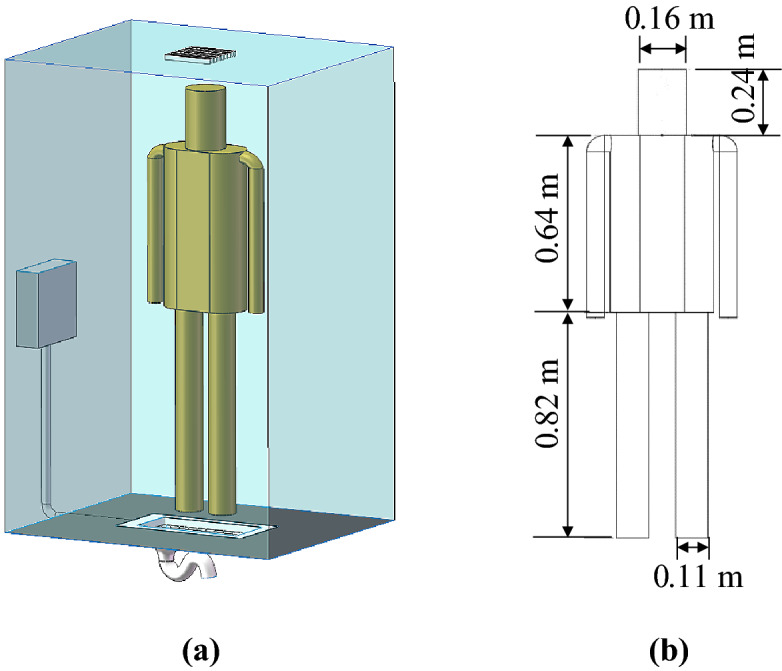
Standing thermal manikin to simulate a lavatory user after toilet flushing: (**a**) picture of the manikin, (**b**) dimensions of the manikin.

A thermal manikin was used to simulate a standing lavatory user remaining inside the lavatory after flushing toilet. As shown in Fig. 11, the manikin (40 cm × 20 cm × 170 cm) included a head, trunk, arms, and legs, but with simplified geometry. The trunk was represented by an elliptical cylinder, and the remaining parts by round cylinders. The manikin’s skin was covered with electric film, which conditioned the surface temperature to 31 °C.

### Flow visualization

Prior to a flushing process, the water mist generated by an ultrasonic atomizer was released into the toilet bowl as a tracer. Because the mist droplets were large, they dropped under the action of gravity. Only a rising airflow would be able to drive the water mist upwards, and thus would evidently indicate a rising toilet plume flow. The lavatory floor, walls, and toilet step area were covered with black sheets to aid viewing of the water mist motion. The water mist was recorded by a cell phone once the flushing button had been pressed. The resolution of the photograph was 1080 × 1920 with a photographing speed of 30 frames per second.

### Transient airflow measurement by particle image velocimetry (PIV)

**Figure 12:**
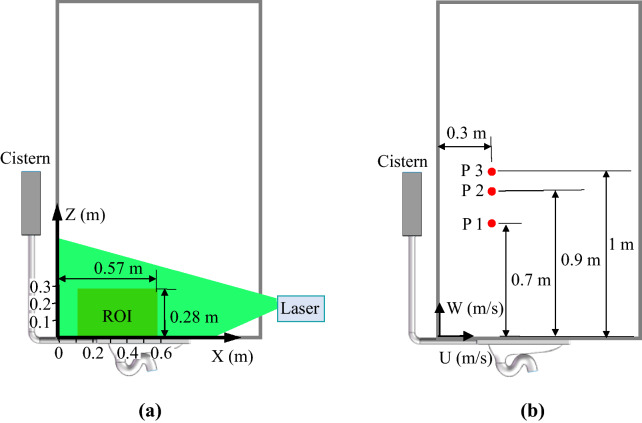
The region or locations for velocity measurement: (**a**) the region of interest (ROI) for the PIV measurement, (**b**) the points for velocity measurement with an ultrasonic anemometer.

A high-power 2D2C-PIV system (Dantec Dynamics, Denmark) was used to measure the transient airflow. The PIV system consisted of a dual-cavity pulsed laser (type: Vlite-Hi-20 k; Beamtech, Canada), a high-speed camera (type: VEO 410L; Phantom, USA) with a resolution of 1280 × 800, a synchronizer (type: 81N21; Dantec Dynamics, Denmark), and a computer. The laser had a pulse time interval of 50 μs at a wavelength of 532 nm. The laser beam passed through a cylindrical lens, forming a light sheet with a thickness of 2 mm in the region of interest (ROI). The image sequence was acquired in the double frame mode, and the time length between pluses was 1500 μs. Diethylhexyl sebacate (DEHS) particles with a mean diameter of approximately 2 μm generated by a monodisperse aerosol generator (type: 3475; TSI, USA) were used as tracer particles. The DEHS particle concentration was in the range of 10–20 particles in an interrogation area of 32 × 32 pixels. Figure 12a shows the ROI in the mid-longitudinal section above the squat toilet. The dimensions of the ROI were 0.46 m × 0.28 m, and the left boundary of the ROI was 0.11 m away from the lavatory wall.

### Transient airflow measurement with an ultrasonic anemometer

In addition to PIV, this study used a three-dimensional ultrasonic anemometer (type: DA650 & TR92T; Kaijo Sonic, Japan) to measure the velocity at specific points. The anemometer had a resolution of 0.005 m/s with 1% uncertainty. The measuring frequency was 20 Hz. Figure 12b shows the three points in the mid-longitudinal section at which the velocities were measured. The measurements in each location were repeated at least 5 times.

### Measurement and inference of particle source strength

**Figure 13:**
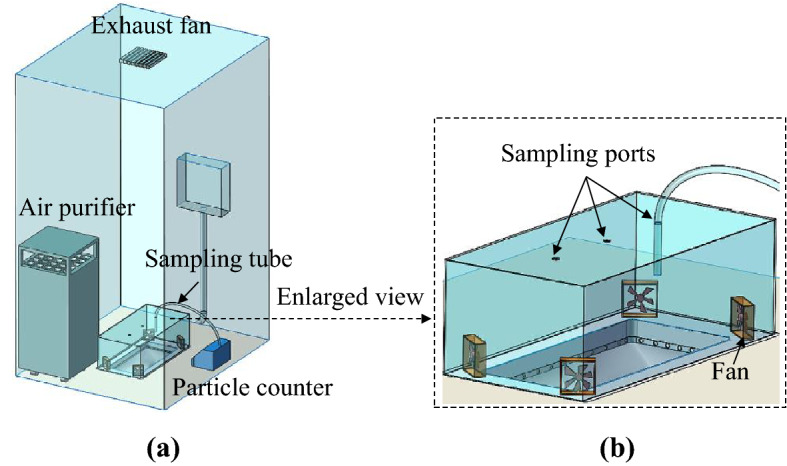
Schematics for measuring the particles inside a box for inference of the particle source: (**a**) view of the whole lavatory, (**b**) enlarged view of the box for particle counting.

As shown in Fig. 13a, the squat toilet was covered by a transparent acrylic box with dimensions of 0.6 m × 0.3 m × 0.2 m for particle concentration measurement. To mix the particles inside the box, four small fans were installed, one in each corner of the box. The well-mixed particle concentrations would be used for the subsequent inference of the particle generation rate. Figure 13b depicts the three sampling ports on the top of the box, through which the sampling tubes could reach the mid-height of the box. A particle counter (type: 9310–02; TSI, USA) was adopted to measure the temporal concentrations in six bins: (i) 0.3–0.5 μm, (ii) 0.5–1 μm, (iii) 1–3 μm, (iv) 3–5 μm, (v) 5–10 μm, and (vi) 10–25 μm. The counting efficiency was 50% for particles at 0.3 μm, while the efficiency reached 100% for particles larger than 0.45 μm. The rated sampling airflow rate was 28.3 L/min with ± 5% accuracy. The particle counter was within one year of calibration when it was used for test. The measuring frequency was 1 Hz.

In order to minimize interruption by background particles, an air purifier was continuously run inside the lavatory. In addition, the squat toilet, lavatory walls and floor, and box were cleaned repeatedly before the experiment to minimize resuspension of deposited particles during the flushing process. The particle concentrations were measured for the case with a flushing operation and for the case without a flushing operation. Each case was repeated at least three times, and the time interval between two successive flushing cases was at least 10 min. The surrounding air temperature during the tests was 24.0 ± 1.0 °C, and the relative humidity was 50% ± 2%.

The generated total number and size-resolved number of particles per flush were determined from the temporal particle concentrations. The main factors contributing to the particle concentrations inside the box included the particles generated by the flush, the particles deposited due to gravity, the particles drawn in from the outside of the box, and the particles drawn out by the particle counter^36^. Taking these factors into account and assuming a well-mixed condition in the box, the governing equation can be written as^37^:

1 $$\frac{{{\text{d}}C(t)}}{{{\text{d}}t}} = \frac{{E_{{\text{r}}} (t)}}{V} + P\alpha C_{{{\text{out}}}} - (k + \alpha )C(t),$$

where *C*(*t*) is the particle concentration in the box at time *t* (particles/L); *C*_out_(*t*) is the particle concentration outside the box (particles/L); *E*_r_(*t*) is the particle emission rate (particles/s) due to the flush; *V* is the volume enclosed by the box and toilet bowl, which was approximately 45.64 L; *P* is the particle penetration efficiency; *k* is the particle deposition rate (s^-1^); *α* is the air exchange rate (s^-1^); and (*k* + *α*) is the total particle removal rate (s^-1^). In Eq. (1), the evaporation, condensation, and collision of particles were neglected.

If there is no flush, by following Eq. (1) the background particle concentration inside the box can be expressed as:

2 $$\frac{{{\text{d}}C_{{{\text{back}}}} (t)}}{{{\text{d}}t}} = P\alpha C_{{{\text{out}}}} - (k + \alpha )C_{{{\text{back}}}} (t).$$

Because the background particle concentration was nearly unchanged when there was no disturbance, the number of particles penetrating into the box from outside was approximately equal to the total number of particles removed, i.e., *PαC*_out_ was equal to (*k* + *α*)*C*_back_(*t*). When Eq. (2) is subtracted from Eq. (1), the net particle concentration in the box due to the flush can be written as:

3 $$\frac{{{\text{d}}C_{{{\text{net}}}} (t)}}{{{\text{d}}t}} = \frac{{E_{{\text{r}}} (t)}}{V} - (k + \alpha )C_{{{\text{net}}}} (t),$$

where *C*_net_(*t*) is the particle concentration in the box at time *t* (particles/L) due to the flush only.

If (*k* + *α*) is assumed to be time independent, Eq. (3) can be integrated into:

4 $$Emissions = V \times [C_{{{\text{net}}}} (t_{{\text{p}}} ) + (k + \alpha )\int_{{t_{{\text{s}}} }}^{{t_{{\text{p}}} }} {C_{{{\text{net}}}} (t){\text{d}}t} ,$$

where *Emissions* is the total number of particles generated per flush, *t*_s_ is the start time of the flushing process, *t*_p_ is the moment when the particle concentration reaches its peak, and *C*_net_(*t*_p_) is the peak particle concentration (particles/L). The term (*k* + *α*) can be obtained by using the concentrations in the descent stage, during which there is no particle emission. With *E*_r_(*t*) = 0 in Eq. (3), the following equation is obtained after integration:

5 $$\ln \frac{{C_{{{\text{net}}}} (t_{{\text{d}}} )}}{{C_{{{\text{net}}}} (t_{{\text{p}}} )}} = - (k + \alpha ) \times (t_{{\text{d}}} - t_{{\text{p}}} ),$$

where *t*_d_ is a moment in the descent stage, and *C*_net_(*t*_d_) is the particle concentration at *t*_d_ (particles/L).

### Particles deposited on key surfaces

This investigation examined the fluorescent droplets/particles deposited on the step area of the squat toilet, the flushing button, and the lavatory door handle. Prior to toilet flushing, 20 g fluorescent powder was added to the cistern and 20 g to the toilet trap. For clear viewing of the deposition of fluorescent particles on the step area of the toilet, a violet light was used to illuminate the floor. The observation of fluorescent particles deposited on the flushing button and door handle was conducted in another test. All the wall surfaces inside the lavatory were thoroughly cleaned to minimize disruption of the tests. A settling plate was placed on the upper surface of the flushing button, and another was placed on the door handle. An inverted fluorescent microscope (type: IX71; Olympus, Japan) was used to photograph the deposited fluorescent particles.

### Exposure evaluated by means of a tracer gas

A tracer gas is a suitable surrogate for droplet nuclei in the built environment^38^. To minimize the buoyancy force caused by the tracer-gas, this investigation used a mixture of of 1% SF_6_ and 99% N_2_. The SF_6_ concentration was measured with a multi-point sampler (type: Innova 1409; LumaSense, Denmark) and an infrared photoacoustic gas monitor (type: Innova 1412i; LumaSense, Denmark). The resolution of the test instruments was 0.01 ppm, and the nominal accuracy was 1%. Although continuous sampling was employed in this investigation, each concentration reading lasted 35 s.

**Figure 14:**
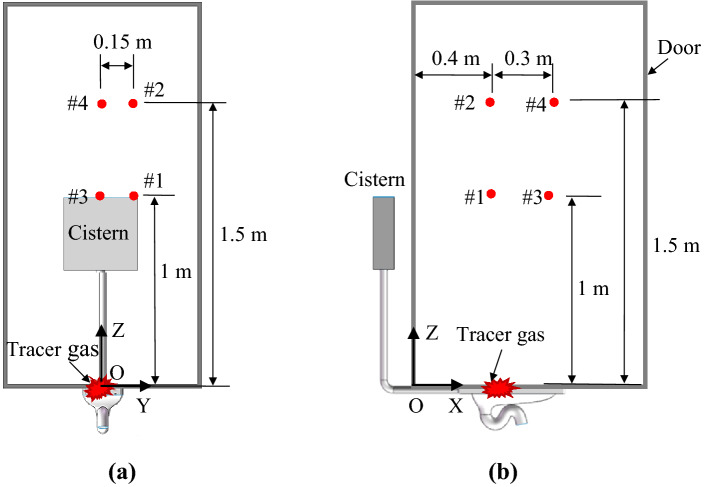
Sampling locations for the SF_6_ tracer gas concentration: (**a**) front view, (**b**) side view.

The tracer gas concentration was measured in different breathing heights, as shown in Fig. 14. The heights of 1 m and 1.5 m above the floor corresponded to the breathing levels of a standing adult and child, respectively. The SF_6_ gas was injected into the toilet bowl at a flow rate of 5 L/min for approximately 50 s prior to the toilet flushing. The toilet was then flushed, and the concentrations at the four points were measured, for a repetition of at least 5 tests. After a test, the exhaust fan was kept running for 2 h to remove the residual SF_6_ gas inside the lavatory before the subsequent test.

### Projected inhaled particle number

Suppose a lavatory user whose breathing zone was located at the four points as shown in Fig. 14. The share ratio of the inhaled SF_6_ gas with respect to the totally released SF_6_ gas could be integrated from the monitored SF_6_ gas concentration as:

6 $$Inhaled\,ratio=\frac{\int {C}_{\mathrm{SF}6}\bullet p\bullet dt\bullet {10}^{-6}}{{\Delta V}_{\mathrm{SF}6}}$$

where *C*_SF6_ is the monitored SF_6_ gas concentrations (ppm) as shown in Fig. 8, *p* is the pulmonary ventilation rate (L/min) of the lavatory user, *t* is the residence time (min) after a flushing process, and ${\Delta V}_{\mathrm{SF}6}$ is the totally released SF_6_ gas (L) into the toilet bowl, which was 0.042 L in this investigation. Assume that the lavatory user had a tidal volume of 0.6 L (the air volume expired in a single breath) and the duration of each breathing cycle was 6 s. Also assume that the inhalation and the exhalation were identical and there was a short break of 0.5 s between them. Then an average inhalation rate of 14.4 L/min for 2.5 s in a breathing cycle would be resulted. Approximating the varying, intermittent respiration into the continuous (without stopping), constant form would lead a steady pulmonary ventilation rate of 6 L/min^39^, which was the *p* used in this investigation.

Suppose that the airborne particles could well trace the SF6 gas, which is mostly the case for small-size droplet nuclei. The inhaled ratio of the SF6 gas and the droplet nuclei would be identical. Then the possibly inhaled particle number could be calculated as the product of the totally generated particle number in toilet bowl and the inhaled ratio in Eq. (6).

## Supplementary Information

Supplementary Information 1.

Supplementary Information 2.

Supplementary Information 3.

Supplementary Information 4.

Supplementary Information 5.

## Data Availability

All data generated or analyzed during this study are included in this published article and its supplementary information files.
