# Supplementary figures and images for: Particle exposure risk to a lavatory user after flushing a squat toilet
Source: Sci Rep. 2022 Dec 6;12:21088. doi: 10.1038/s41598-022-25106-4 (PMC9726816; doi:10.1038/s41598-022-25106-4)

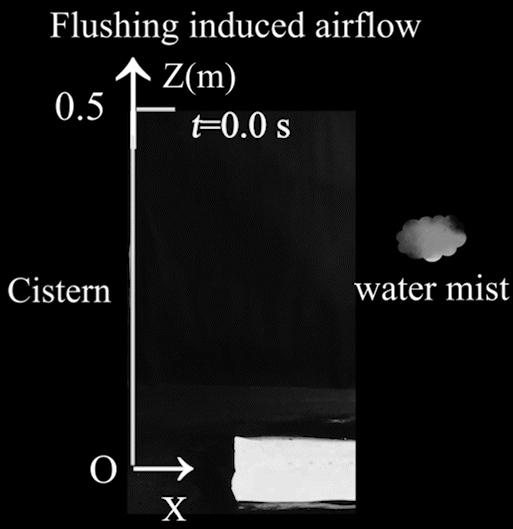

Supplement: Supplementary file 1 — Supplementary Information 1. [file 41598_2022_25106_MOESM1_ESM.gif]

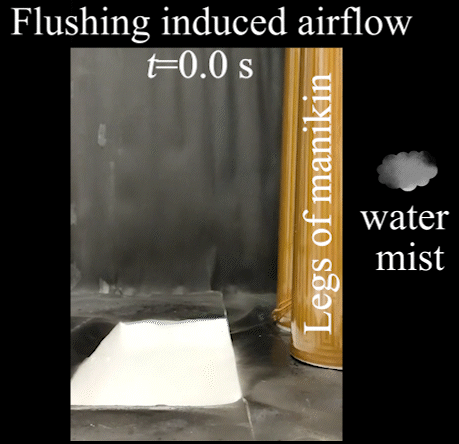

Supplement: Supplementary file 2 — Supplementary Information 2. [file 41598_2022_25106_MOESM2_ESM.gif]

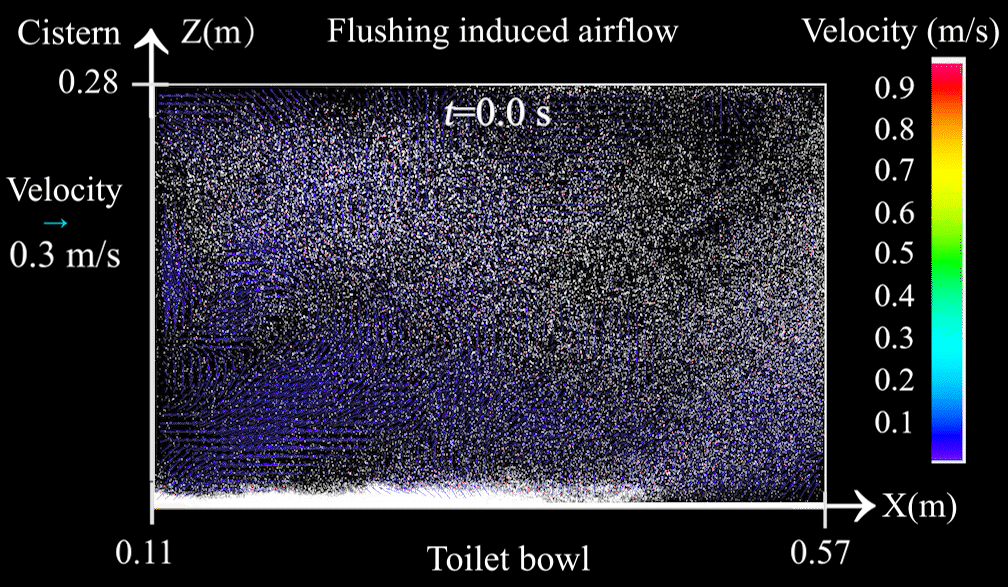

Supplement: Supplementary file 3 — Supplementary Information 3. [file 41598_2022_25106_MOESM3_ESM.gif]

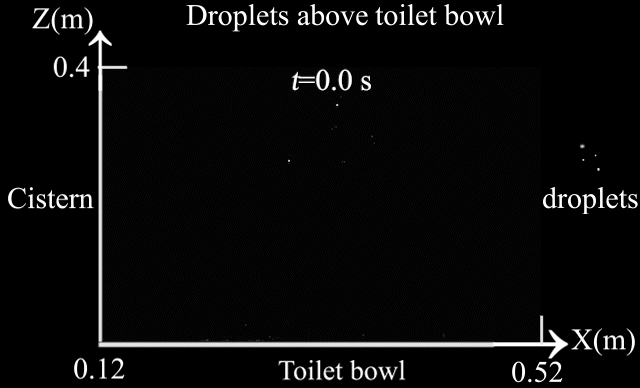

Supplement: Supplementary file 4 — Supplementary Information 4. [file 41598_2022_25106_MOESM4_ESM.gif]
