# Supplementary material for: Particle exposure risk to a lavatory user after flushing a squat toilet
Source: Sci Rep. 2022 Dec 6;12:21088. doi: 10.1038/s41598-022-25106-4 (PMC9726816; doi:10.1038/s41598-022-25106-4)
Supplement: Supplementary file 5 — Supplementary Information 5. [file 41598_2022_25106_MOESM5_ESM.docx]

**Supporting Information for**

Particle exposure risk to a lavatory user after flushing a squat toilet

Tengfei (Tim) Zhang^1^, Lifang Yao^1^, Zilong Gao^1^, Feng Wang^2,*^

^1^ School of Civil Engineering, Dalian University of Technology, Dalian, China

^2^ Tianjin Laboratory of Indoor Air Environmental Quality Control, School of Environmental Science and Engineering, Tianjin University, Tianjin, China

*^*^Corresponding email: f.wang@tju.edu.cn*

**Analysis of PIV measurement accuracy**

The air velocities at two typical points measured by the PIV system and the ultrasonic anemometer were compared. Fig. S.1 presents the transient air speeds at two points, i.e., (0.3 m, 0 m, 0.05 m) and (0.3 m, 0 m, 0.1 m). In general, the two methods provided air speeds that were in good agreement with each other. There was only a small difference between them, mainly due to the repeatability of the flushing process and the different time frequencies for sensing the airflow.

| 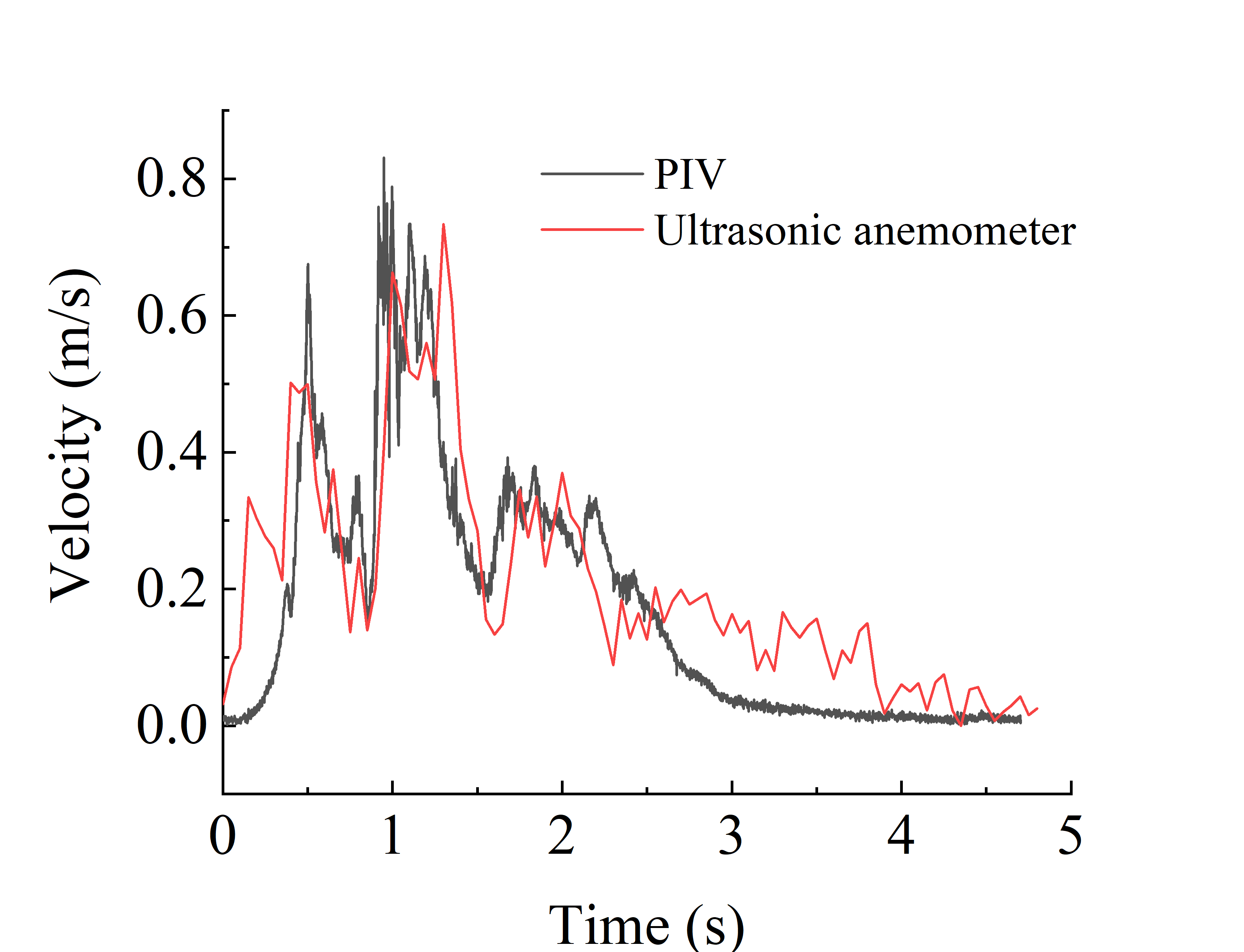 Time (s)  0.2  0.0  0.4  0.6  0.8  Velocity (m/s)  PIV  Ultrasonic anemometer  0  1  2  3  4  51 | 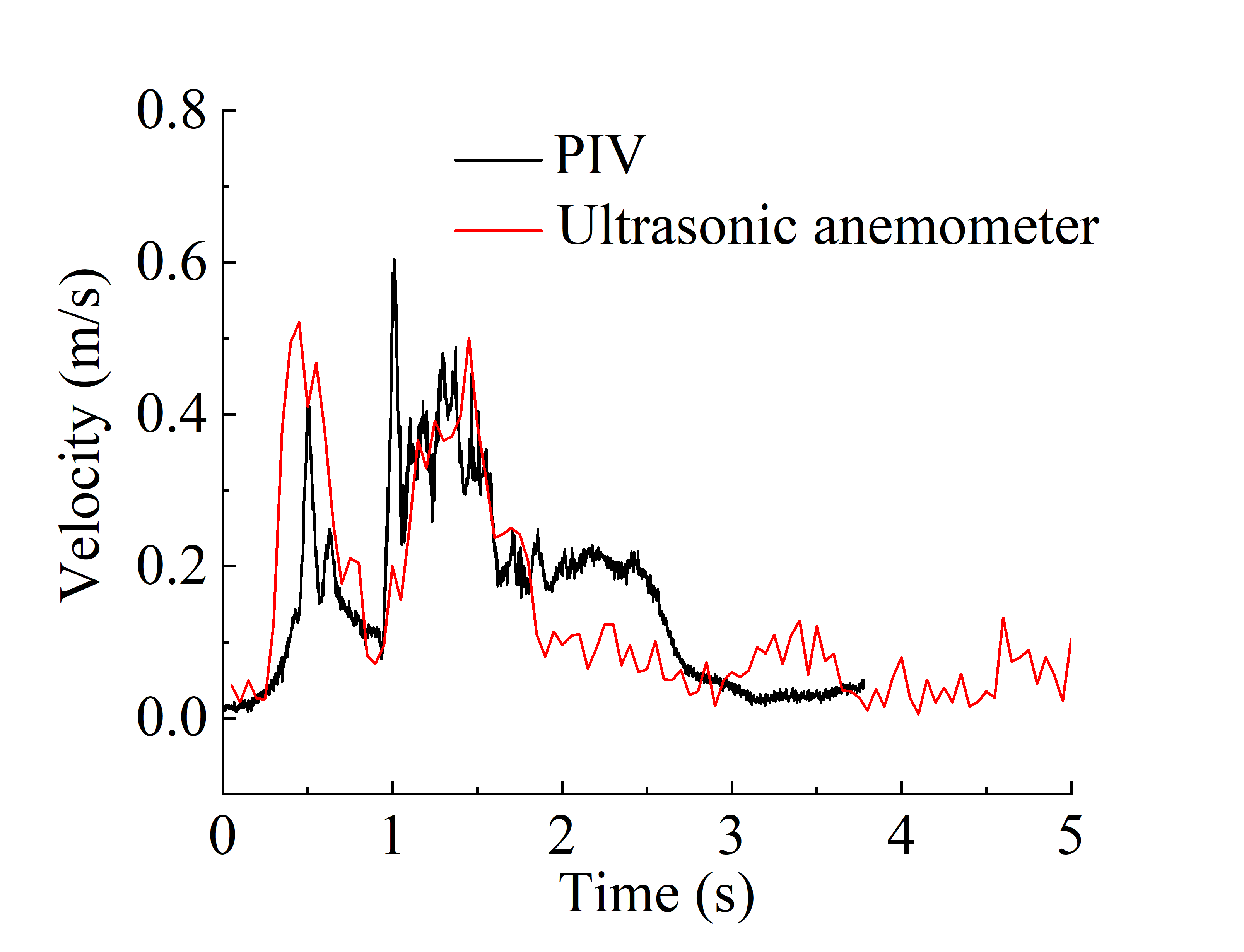 0.2  0.0  0.4  0.6  0.8  Velocity (m/s)  Time (s)  0  1  2  3  4  5  PIV  Ultrasonic anemometer |
| --- | --- |

(a) (b)

**Fig. S.1.** Comparison of the omnidirectional velocity measured by PIV and ultrasonic anemometer: (a) at point (0.3 m, 0 m, 0.05 m), (b) at point (0.3 m, 0 m, 0.1 m).

| *UncR* (m/s) 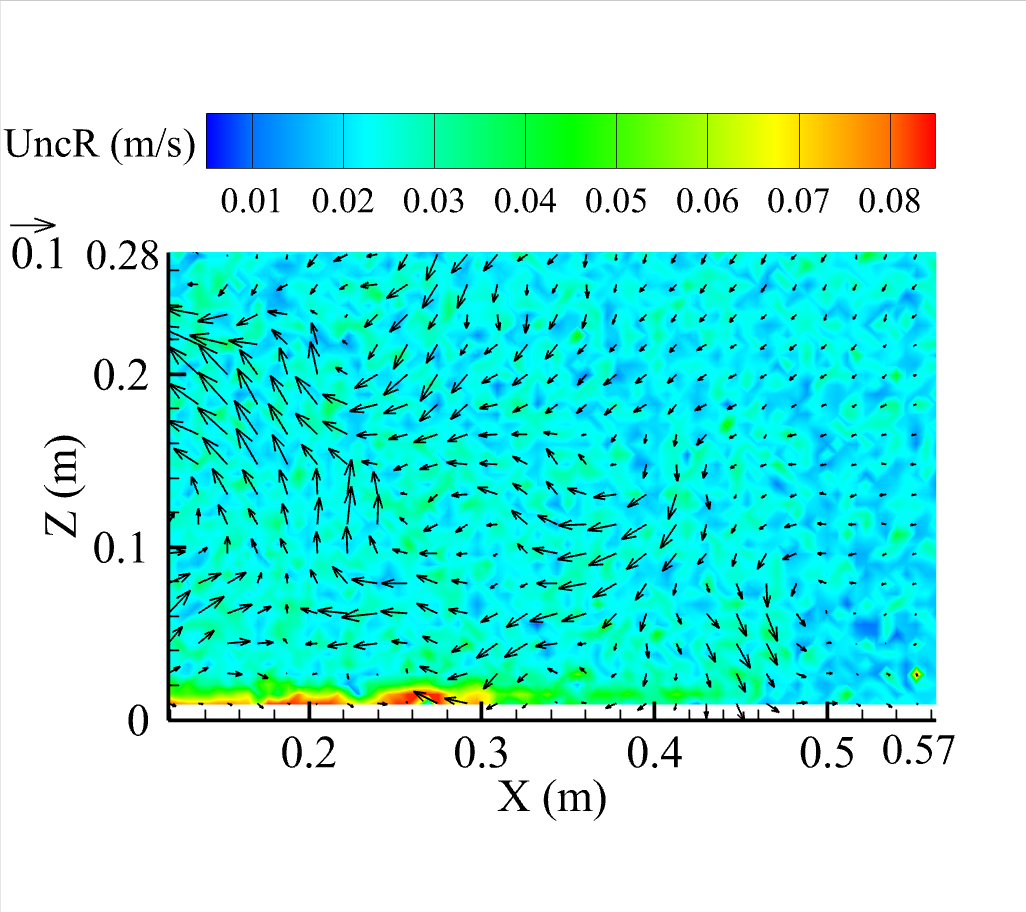 0.01  0.02  0.03  0.04  0.05  0.06  0.07  0.08 | |
| --- | --- |
| 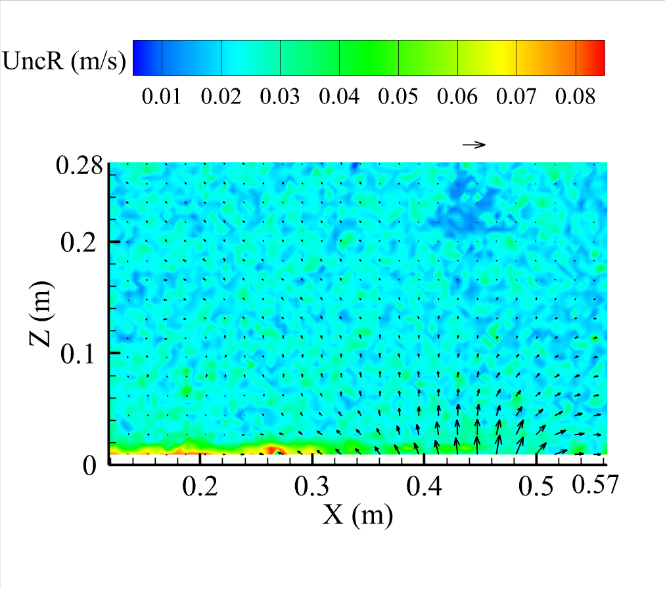 0  0.1  0.2  Z (m)  0.28  0.2  0.3  0.4  0.5  0.11  X (m)  0.2 m/s | 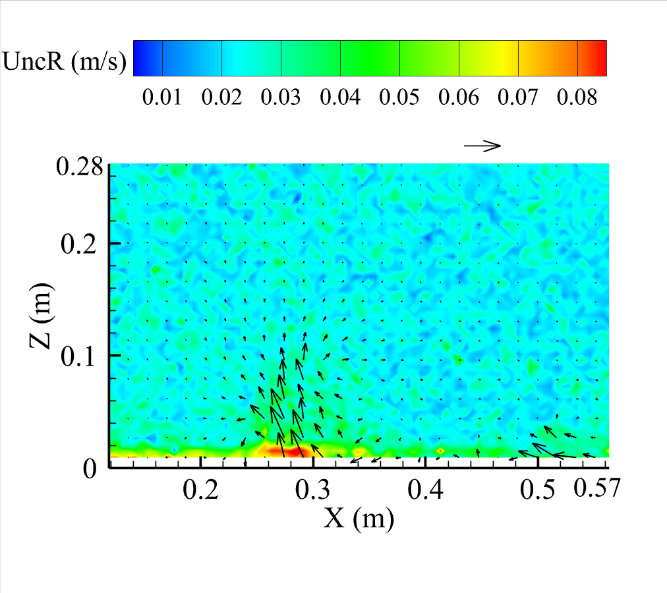 0  0.8 m/s  Z (m)  0.1  0.2  0.28  0.2  0.3  0.4  0.5  0.11  X (m) |
| (a) | (b) |
| 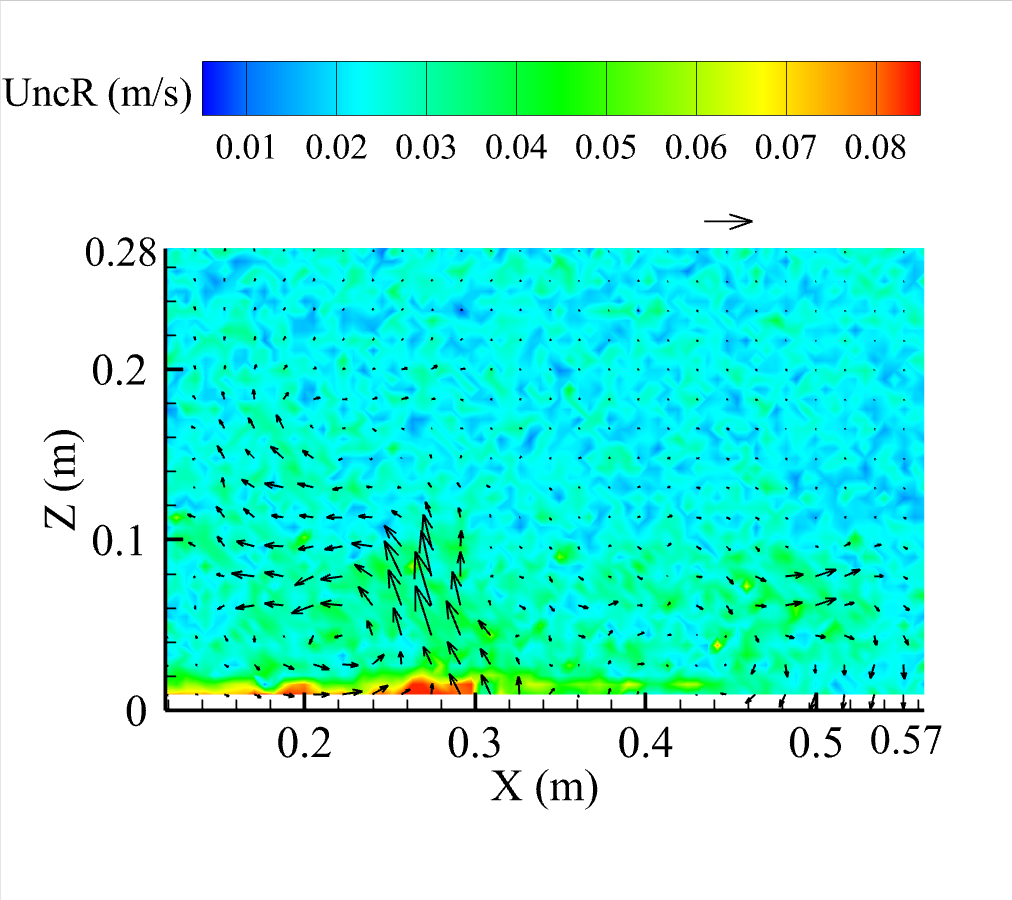 0  0.7 m/s  Z (m)  0.1  0.2  0.28  0.2  0.3  0.4  0.5  0.11  X (m) | 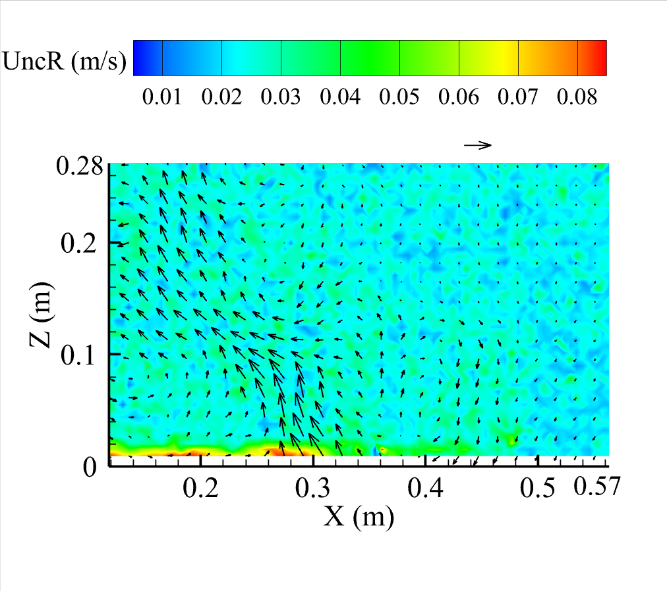 0  0.3 m/s  Z (m)  0.1  0.2  0.28  0.2  0.3  0.4  0.5  0.11  X (m) |
| (c) | (d) |
| 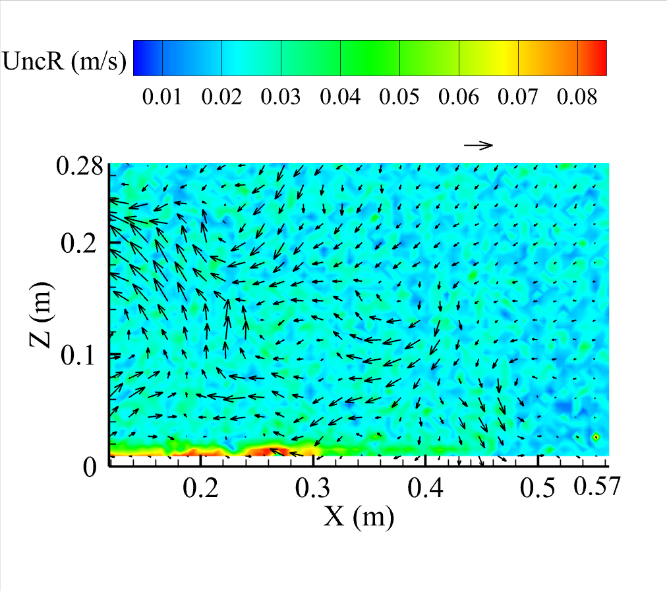 0  0.1 m/s  Z (m)  0.1  0.2  0.28  0.2  0.3  0.4  0.5  0.11  X (m) | 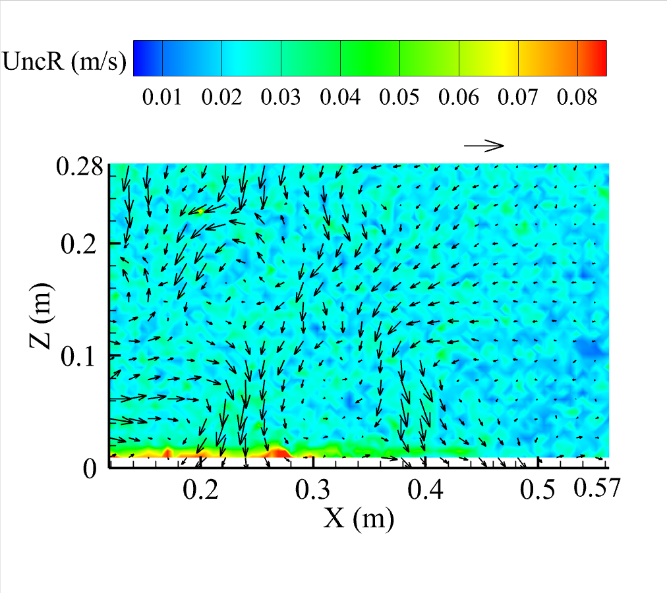 0  0.1 m/s  Z (m)  0.1  0.2  0.28  0.2  0.3  0.4  0.5  0.11  X (m) |
| (e) | (f) |

**Fig. S.2.** The radial error of the measured air velocity field by PIV (*UncR*) for a flushing process: (a) at *t* = 0.2 s, (b) at *t* = 0.5 s, (c) at *t* = 1.0 s, (d) at *t* = 2.0 s, (e) at *t* = 3.0 s, (f) at *t* = 4.0 s.

The uncertainties of the measured velocities by PIV were also analyzed. The particle disparity quantification proposed by Sciacchitano [S1] was adopted. The velocity uncertainties were estimated using the velocity field itself as input along with the original particle images. The particle disparity technique identified individual particles on two consecutive frames and tried to match pairs based on the measured displacement vectors. The standard deviation *σ* of the disparity vector was used to quantify the magnitude of the random errors and was referred as the “uncertainty.” The particle disparity technique could output uncertainties for *U*, *V*, and the radial error (*UncR*), respectively. The radial error (*UncR*) was the root square sum of the uncertainties of both *U* and *V*. In this study, the confidence interval was 39% for the measured velocities deviating from the actual value by no more than *σ*. Fig. S.2 shows the uncertainties of the measured air velocities, mostly in the range of 0.02–0.05 m/s. The largest uncertainty was less than 0.08 m/s. It implied that the PIV system could obtain the velocity distribution with relatively good accuracy.

**Animation of the flows and generated droplets**

A total of four animations in gif format are provided in this article as supporting information, as shown in Table S.1. Interesting readers may view the animations online.

**Table S.1.** Overview of the animations to show the dynamic flows and generated droplets after flushing a squat toilet.

| **No.** | **Title** | **Legend** |
| --- | --- | --- |
| 1 | Flushing induced airflow visualized by water mist (side view) | The flushing induced airflow is viewed from the lavatory side. The cistern is on the left hand side, while the lavatory door is on the right hand side. |
| 2 | Flushing induced airflow visualized by water mist (front view) | The flushing induced airflow is viewed in the direction from the lavatory door to the cistern. |
| 3 | Flushing induced airflow above toilet bowl by PIV | The flushing induced airflow above toilet bowl lasts for four seconds. The cistern is on the left hand side, while the lavatory door is on the right hand side. The flushing water rinses the toilet bowl from the right to the left. |
| 4 | Generated droplets above toilet bowl after flushing toilet | The generated droplets above toilet bowl lasts for four seconds. The cistern is on the left hand side, while the lavatory door is on the right hand side. The flushing water rinses the toilet bowl from the right to the left. |

# REFERENCE

[S1] A. Sciacchitano, B. Wieneke, F. Scarano, PIV uncertainty quantification by image matching, Meas. Sci. Technol. 24 (4) (2013) 045302.
